# Elucidation of the mechanism of subunit exchange in αB crystallin oligomers

**DOI:** 10.1038/s41598-021-82250-z

**Published:** 2021-01-28

**Authors:** Rintaro Inoue, Yusuke Sakamaki, Takumi Takata, Kathleen Wood, Ken Morishima, Nobuhiro Sato, Aya Okuda, Masahiro Shimizu, Reiko Urade, Noriko Fujii, Masaaki Sugiyama

**Affiliations:** 1 Institute for Integrated Radiation and Nuclear Science, Kyoto University, Kumatori, Sennan-gun, Osaka 590-0494 Japan; 2 Australian Nuclear Science and Technology Organization, Lucas Heights, NSW Australia

**Keywords:** Biological techniques, Biophysics

## Abstract

AlphaB crystallin (αB-crystallin) is a key protein for maintaining the long-term transparency of the eye lens. In the eye lens, αB-crystallin is a “dynamical” oligomer regulated by subunit exchange between the oligomers. To elucidate the unsettled mechanism of subunit exchange in αB-crystallin oligomers, the study was carried out at two different protein concentrations, 28.5 mg/mL (dense sample) and 0.45 mg/mL (dilute sample), through inverse contrast matching small-angle neutron scattering. Interestingly, the exchange rate of the dense sample was the same as that of the dilute sample. From analytical ultracentrifuge measurements, the coexistence of small molecular weight components and oligomers was detected, regardless of the protein concentration. The model proposed that subunit exchange could proceed through the assistance of monomers and other small oligomers; the key mechanism is attaching/detaching monomers and other small oligomers to/from oligomers. Moreover, this model successfully reproduced the experimental results for both dense and dilute solutions. It is concluded that the monomer and other small oligomers attaching/detaching mainly regulates the subunit exchange in αB-crystallin oligomer.

## Introduction

α-crystallin, which is a major protein in the eye lens, contributes to the long-term transparency of the eye lens due to its chaperone activity^1^. It is known that α-crystallin is an oligomer composed of 20–40 subunits^2^, and two types of subunits, αA and αB, serve as building components of the oligomer. To unveil the mechanism of its chaperone activity, the quaternary structure of the α-crystallin oligomer has been tackled by state-of-the-art experimental techniques. However, the structure has not been solved, and even the association number, *n*, has been dispersed depending on the reports (*n* = 20–32)^3–7^. To explain this chaotic situation, it has been hypothesized that the α-crystallin oligomer intrinsically lacks a “static” quaternary structure. To be more specific, their association numbers are not fixed, and their corresponding quaternary structures fluctuate dynamically because α-crystallin oligomers exchange subunits between them. van den Oetelaar et al.^8^ firstly suggested the existence of subunit exchange in α-crystallin utilizing reaggregated bovine α-crystallin. After their pioneering work, some groups have studied the subunit exchange in α-crystallin under several experimental conditions through fluorescence resonance energy transfer^9,10^. Baldwin et al.^11^ have also studied the subunit exchange in αB-crystallin through solution nuclear magnetic resonance (NMR). Despite of the extensive works on the subunit exchange in α-crystallin, the final consensus concerning the mechanism of subunit exchange in α-crystallin has not been reached. It is expected that elucidation of the mechanism of subunit exchange must contribute to unveiling the mechanism of its chaperone activity. Motivated by such an idea, we commenced to investigate the subunit exchange in homo-oligomer of αB-crystallin with deuteration-assisted small-angle neutron scattering (DA-SANS) technique^12^. We succeeded in proving the existence of this subunit exchange from the time dependence of forward scattering intensity (*I*_0_)^13^.

As a next step, we then aimed to elucidate the subunit exchange mechanism. One of the possible models is that the subunit exchange proceeds through a collision between the oligomers. Under this model, an increase in the exchange rate is observed with protein concentration because a higher protein concentration increases the frequency of collisions. This model must be examined by observing the exchange rate as a function of protein concentration. Since our previous study was performed using a dilute protein concentration^13^, we should study the subunit exchange at higher protein concentrations. Here, it evokes an inherently difficult problem: an increase in protein concentration accompanies inter-particle interference, especially in the low *Q* region, hindering the accurate determination of *I*_0_. To overcome this problem, we should develop a new experimental approach that enables free inter-particle interference, even at high protein concentrations.

One promising technique is inverse-contrast matching SANS (iCM-SANS)^14,15^. It makes partially (approximately 75%)-deuterated (*p*d-) protein “scatteringly invisible” in D_2_O through matching the scattering length density (SLD) of a 75d-protein to that of D_2_O. In other words, a hydrogenated (h-) protein is “scatteringly visible” and *p*d-protein is “scatteringly invisible” in D_2_O. Accordingly, when “dilute” h-proteins and “dense” *p*d-proteins are mixed in D_2_O, it is possible to exclusively observe the scattering from the “dilute” h-proteins without inter-particle interference under high protein concentrations.

In this study, we applied the iCM-SANS technique to study subunit exchange in αB-crystallin oligomers at two different protein concentrations. One is a dense sample, which consists of dense “scatteringly invisible” *p*d-αB-crystallin oligomer and dilute “scatteringly visible” h-αB-crystallin oligomer. The other is a dilute sample, which consists of dilute “scatteringly invisible” *p*d-αB-crystallin oligomer and dilute “scatteringly visible” h-αB-crystallin oligomer. Based on the experimental results, we propose a reasonable model for the mechanism of subunit exchange in αB-crystallin oligomers.

## Results and discussion

### Monitoring of subunit exchange through iCM-SANS

We first prepared two types of oligomers: one fully consisted of *p*d-subunits, and the other was composed of h-subunits. Here, we denote the oligomer with *m* from *p*d-subunits as OLG(*m*) and, with this notation, the first as OLG(26) and the last as OLG(0) since the average association number of the αB-crystallin oligomer was found to be 26 in our previous work^13^. It was observed that dilute OLG(26) (0.45 mg/mL) was nearly invisible in the full D_2_O buffer (99.6% D_2_O ratio) (Fig. S1).

Next, we set the target protein concentration of αB-crystallin oligomers in the dense system to ~ 30 mg/mL, which is similar to that found in the eye lens of human babies (~ 30 mg/mL)^16^. To overcome the inter-particle interference in such a dense system, our system was composed of dense OLG(26) (28.5 mg/mL) and dilute OLG(0) (0.45 mg/mL) in D_2_O. Even though OLG(26) was dense, they are, in principle, “scatteringly invisible,” and it was expected that the dilute OLG(0) would be exclusively observed without inter-particle interference, even in the dense system. To confirm this, the scattering visibilities for both the dense OLG(26) and the dilute OLG(0) were observed using iCM-SANS. Figure S2 shows the SANS profiles. It was found that the dense OLG(26) was nearly “scatteringly invisible” in D_2_O. In addition, we should emphasize that the scattering intensity of the dilute OLG(0) was approximately four times higher than that of the dense OLG(26) around 0.01 Å^−1^, supporting that the dilute OLG(0) is exclusively observable in the dense system.

**Figure 1:**
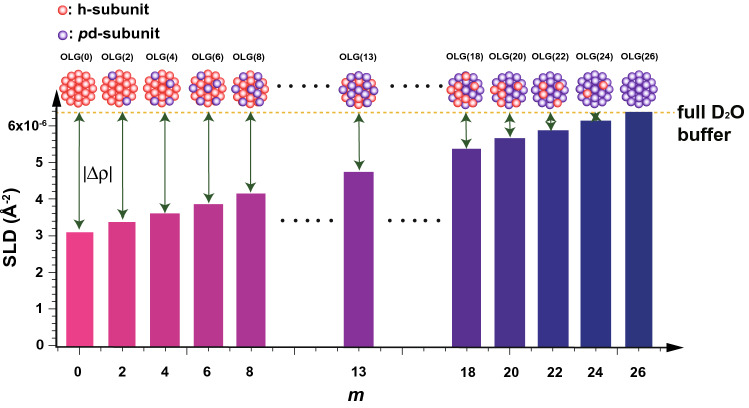
SLDs of OLG(*m*) (bars) and full D_2_O buffer (yellow dotted line). The pink and purple spheres correspond to the h- and *p*d-subunits, respectively. The double-sided arrows indicate the absolute values of the scattering contrast (|Δ*ρ*|). This figure is prepared by the usage of IGOR Pro 6.34A (https://www.wavemetrics.com/forum/news-and-announcements/igor-634a-now-shipping) and Adobe Illustrator CC 2015.2.1 (19.2.1) (https://www.adobe.com/jp/products/illustrator.html).

An explanation of how to monitor subunit exchange with iCM-SANS follows. The dense system (~ 30.0 mg/mL) is created with a mixture of OLG(26) (28.5 mg/mL) and OLG(0) (0.45 mg/mL). When subunit exchange occurs, the oligomers, including the *p*d- and h-subunits, such as OLG(*m*) (0 < *m* < 26), are gradually generated, and finally, the distribution of OLG(*m*)s reaches the equilibrium state. Here, the time evolution of the SANS intensity is measured, especially the forward scattering intensity, *I*_0_, just after mixing *I*_0_ is expressed by the following equation:

1 $$I_{0} = NV^{2} (\rho_{{{\text{protein}}}} - \rho_{{{\text{solvent}}}} )^{2} = NV^{2} \Delta \rho^{2} ,$$

where *N*, *V*, *ρ*_protein_, and *ρ*_solvent_ correspond to the number density, volume, and SLDs of protein and solvent, respectively, and Δ*ρ* (= *ρ*_protein_ − *ρ*_solvent_) is the “scattering contrast”. Figure 1 shows the SLDs of OLG(*m*) and full D_2_O buffer (99.6% D_2_O ratio), respectively, and |Δ*ρ*| of OLG(*m*), which is proportional to (26 − *m*), is indicated by the length of the double-sided arrows. As given in Eq. (1), *I*_0_ is proportional to the square of Δ*ρ*. *I*_0_ starts to decrease with the progress of subunit exchange until reaching the equilibrium state.

**Figure 2:**
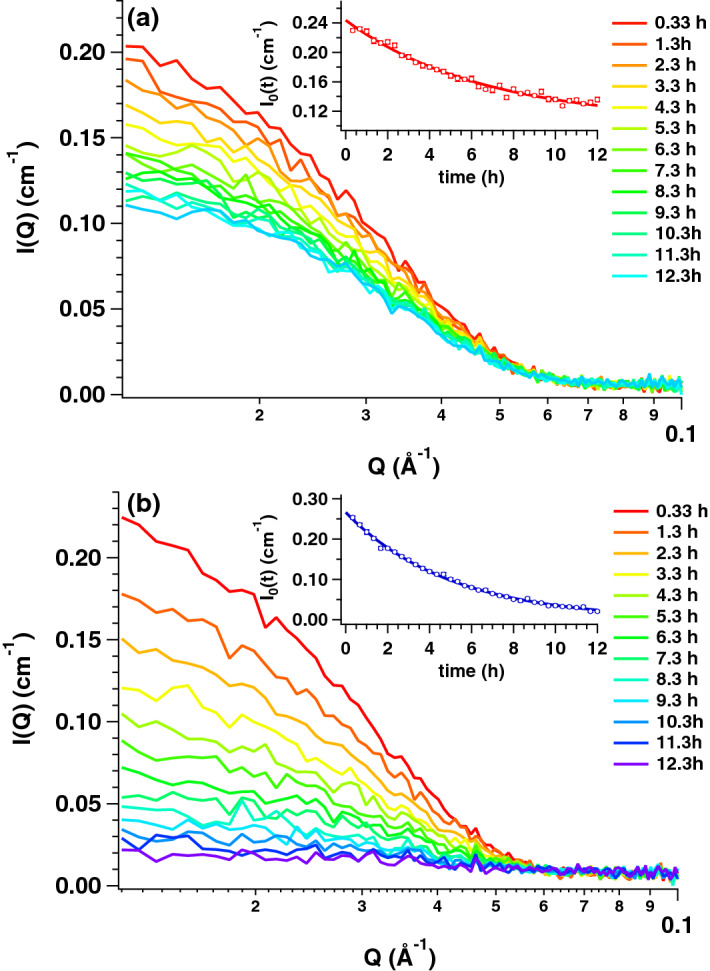
Time evolutions of the SANS profile after being mixed with OLG(0) and OLG(26) for the dilute and dense samples. (**a**) Time evolution of the SANS profile after being mixed with OLG(0) at 0.45 mg/mL and OLG(26) at 0.45 mg/mL (dilute sample) at 37 °C (red to light blue lines correspond to the SANS profile at 0.33 h and to that at 12.3 h). The inset indicates *I*_0_(*t*), and the red solid line corresponds to the fit with Eq. (2). (**b**) Time evolution of the SANS profile after being mixed with OLG(0) at 0.45 mg/mL and OLG(26) at 28.5 mg/mL (dense sample) at 37 °C (red to purple lines correspond to the SANS profile at 0.33 h and to that at 13.3 h). Insets indicate *I*_0_(*t*), and the blue solid line corresponds to the fit with Eq. (2). This figure is prepared by the usage of IGOR Pro 6.34A (https://www.wavemetrics.com/forum/news-and-announcements/igor-634a-now-shipping).

In the present study, we performed time-resolved iCM-SANS experiments with two samples: the mixture of OLG(26) (28.5 mg/mL) and OLG(0) (0.45 mg/mL) as the *dense* sample (~ 30.0 mg/mL) and the mixture of OLG(26) and OLG(0) both with 0.45 mg/mL as the *dilute* reference sample. Figure 2a,b show the time evolutions of the SANS profiles of the dilute and dense samples after mixing. In order to track the time dependence of *I*_0_ (*I*_0_(*t*)), we performed Guinier analyses for both dilute and dense samples. The Guinier plots from both dilute and dense samples are plotted in Fig. S3 and clear decrease of intensity at the low *Q* region were observed for both samples with the progress of time. Fig. S4 shows the time dependence of radius of gyration (*R*_g_) from both dilute and dense samples and *R*_g_ (= 51.9 ± 1.2 Å) of OLG(0) at the concentration of 0.45 mg/mL measured by small-angle X-ray scattering (SAXS) is also included in Fig. S4. Just after mixing of OLG(0) and OLG(26), *R*_g_ of dilute and dense samples coincided with that determined by SAXS within experimental error. This finding supports the absence of inter-particle interference and the validity of evaluation of both *R*_g_ and *I*_0_ in present *Q* range. We then focused on *I*_0_(*t*) from both samples to figure out the progress of subunit exchange and inset figures of Fig. 2 show the time evolutions of the forward scattering intensity, *I*_0_(*t*). Both samples reached equilibrium states at around 12 h after mixing. For the evaluation of the exchange rate *Γ*, the following single decay function was applied.

2 $$I_{0} \left( t \right) \, = I_{0} \left( 0 \right)[A + \, \left( {1 \, - A} \right) \, \exp ( - \Gamma t)],$$

where *A* corresponds to the ratio of the intensity at the equilibrium state to the initial intensity. Both *I*_0_(*t*) values were well fitted with Eq. (2), as shown by the solid lines in the inset figures. *A* and *Γ* were calculated to be 0.50 ± 0.02 and 0.21 ± 0.02 h^−1^ for the dilute sample, and 0.05 ± 0.02 and 0.20 ± 0.02 h^−1^ for the dense sample, respectively. Assuming that every subunit has the same exchangeability, *A* values for the dilute and dense samples were calculated to 0.52 and 0.053, respectively (refer to Supplementary Information and Fig. S5). This means that the subunits were completely exchanged in both samples. It was reported that the dimers serve as assembling components for oligomer of small heat shock protein^17^. To consider the possibility that dimers serve as an exchange unit in αB-crystallin, we also calculated the *A* values (*A*_d_) for the dilute and dense samples under the assumption that every dimer in αB-crystallin oligomer possess the same exchangeability. The *A*_d_ values for the dilute and dense samples were calculated to 0.52 and 0.091, respectively. Especially, it was revealed that *A*_d_ value from the dense sample deviated from experimentally calculated *A* value (= 0.05 ± 0.02) beyond the experimental error. From our previous work, the monomer was mainly observed in low *m*/*z* region from αB-crystallin at 37 °C^13^. In addition, Aquillina et al.^5^ concluded that monomers serve as assembling components of αB-crystallin oligomer. It is then considered that monomers are candidate for the exchanging units in the subunit exchange of αB-crystallin.

The most stressing point was that the exchange rate of the dense sample was the same as that of the dilute sample within experimental error. We firstly assumed that subunit exchange could occur through a random collision between two αB-crystallin oligomers, however this model is excluded as an appropriate model because it cannot explain the subunit exchange mechanism with the observed experimental data (Refer to collision model and Fig. S6, S7 in the Supplementary Materials).

### Detection of small molecular weight components using analytical ultracentrifugation measurements

**Figure 3:**
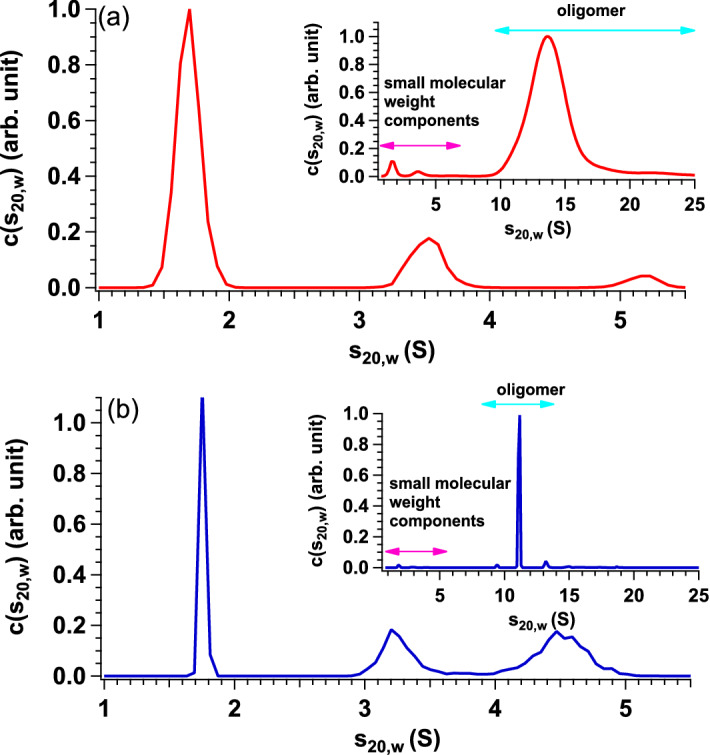
AUC spectra of dilute and dense samples. (**a**) *c*(*s*_20,w_) from αB-crystallin in solution at 0.45 mg/mL measured at 60,000 rpm at 37 °C. The *M*_w_s of three peaks correspond to 19 kDa, 56 kDa and 101 kDa from the low *c*(*s*_20,w_) to the high one, respectively. The inset shows *c*(*s*_20,w_) from αB-crystallin in the solution at 0.45 mg/mL measured at 40,000 rpm at 37 °C. The pink and light blue arrows indicate the region corresponding to small molecular weight components and oligomers, respectively. (**b**) *c*(*s*_20,w_) from αB-crystallin in solution at 28.5 mg/mL measured at 60,000 rpm at 37 °C. *M*_w_s of three peaks correspond to 21 kDa, 53 kDa, and 87 kDa from the low *c*(*s*_20,w_) to the high one, respectively. The inset shows *c*(*s*_20,w_) from αB-crystallin in the solution at 28.5 mg/mL measured at 40,000 rpm at 37 °C. The pink and light blue arrows indicate the region corresponding to small molecular weight components and oligomers, respectively. This figure is prepared by the usage of IGOR Pro 6.34A (https://www.wavemetrics.com/forum/news-and-announcements/igor-634a-now-shipping).

In our previous work with a dilute sample of ~ 1 mg/mL through native mass spectrometry measurements, the coexistence of oligomers and monomers were observed^13^. Although native mass spectrometry is a powerful technique for unveiling the particle distribution as a function of the molecular weight even in the polydisperse system, it is not applicable for dense protein solutions such as αB-crystallin solution at 28.5 mg/mL. To overcome this technical limitation, we performed analytical ultracentrifugation (AUC) for αB-crystallin in solution at 0.45 and 28.5 mg/mL. The insets of Fig. 3a,b show the distributions of the sedimentation coefficient (*c*(*s*_20,w_)) of αB-crystallin in the solutions at 0.45 and 28.5 mg/mL measured at 40,000 rpm, respectively. Although the dense sample was affected by non-ideality due to its high concentration^18^, the minor peaks corresponding to small molecular weight component were observed at low *s*_20,w_ region. In order to observe the small molecular weight component accurately, we also performed AUC measurements at 60,000 rpm of which condition is fitted to the detection of small molecular weight contribution. Results of sedimentation velocity analysis of dilute and dense samples are plotted in Fig. S8 and both data were successfully analyzed without exhibiting systematic error. Sedimentation coefficient distributions from the dilute and dense samples measured at 60,000 rpm are plotted in the main panel of Fig. 3 and clear peaks are observed from both samples. We also calculated the *M*_w_ of each peak in Fig. 3a,b from the conversion of *s*_20,w_ value to *M*_w_ (Fig. S9). For the dilute sample, it was calculated to 19 kDa, 56 kDa, 101 kDa from the low *c*(*s*_20,w_) to high one. As for the dense sample, it was calculated to 21 kDa, 53 kDa, 87 kDa from the low *c*(*s*_20,w_) to high one. Taking into consideration of result from native mass spectrometry measurement^13^ and amino acid sequence of αB-crystallin, the main peak from both dilute and dense samples measured at 60,000 rpm is considered to be monomer.

The weight ratios of the small molecular weight components to the oligomer at 0.45 and 28.5 mg/mL were 0.015 ± 0.002 and 0.015 ± 0.003, respectively. It should be noted that the population of small molecular weight components was not affected within the present protein concentration range (< 28.5 mg/mL).

### Monomer attaching/detaching model

**Figure 4:**
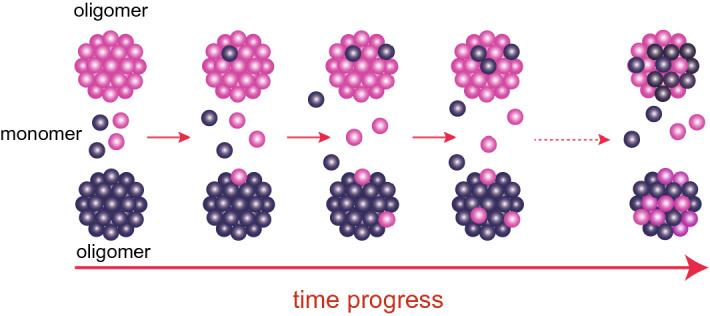
Schematic of the Monomer Attaching/Detaching model. Pink and purple spheres indicate the h- and *p*d-subunits, respectively. Large clusters and small spheres correspond to αB-crystallin oligomers and monomers, respectively. This figure is prepared by the usage of Adobe Illustrator CC 2015.2.1 (19.2.1) (https://www.adobe.com/jp/products/illustrator.html).

Considering the AUC results, it is assumed that the monomer detaches from the oligomer. However, the detached monomer should attach to the oligomer to secure its solubility because the constant exposure of the hydrophobic region of αB-crystallin to solvent is not favourable in terms of solubility^19^. It is then considered that such attached/detached monomers would contribute to the subunit exchange in the αB-crystallin oligomer (refer to Fig. 4). We then introduce the event of monomer attaching/detaching into the second model for subunit exchange in the αB-crystallin oligomer. Based on this new model, *I*_0_(*t*) was calculated using the following procedure.

1. We defined the rates of attaching and detaching monomers as *k*_a_ and *k*_d_, respectively.
2. The distribution of the association number of the αB-crystallin oligomer in the model system reproduced the AUC result (refer to Fig. S10). To simplify the calculation, only the monomer was taken into consideration for the small molecular weight components.
3. To preserve the weight ratio of the small molecular weight components to the oligomer, the ratio of *k*_a_ to *k*_d_ was determined to be 8.6.
4. The number of αB-crystallin oligomers with the association number of *na*, *i* of *p*d-subunits at the calculation step of *cs* was defined as *N*_*na*_[*i*](*cs*) (0 ≤ *i* ≤ *na*). Then, *N*_*na*_[*i*](*cs*) was calculated for each *cs*.
5. Normalized *I*_0_ as a function of *cs* (*I*_0, nor_(*cs*)) was calculated from the distribution of *N*_*na*_[*i*](*cs*).
6. To convert the *I*_0, nor_(*cs*) to *I*_0_(*t*)*,* the time scaling factor was determined by reproducing *I*_0_(*t*).

**Figure 5:**
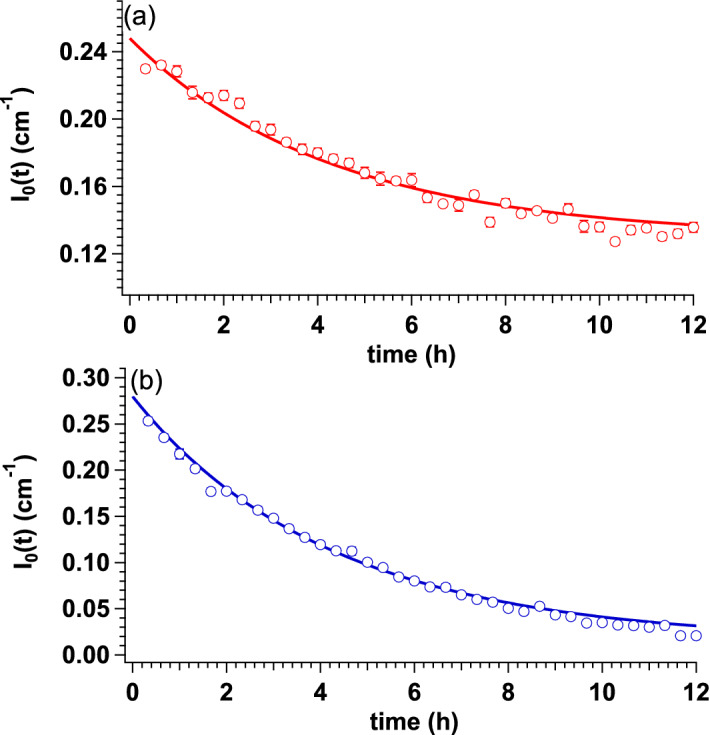
*I*_0_(*t*)s of the dilute and the dense samples, and the calculated curves based on the monomer attaching/detaching model. (**a**) The red circle and the red solid line correspond to *I*_0_(*t*) of the dilute sample and the result from the calculation based on the Monomer Attaching/Detaching model, respectively. (**b**) The blue circle and the blue solid line correspond to *I*_0_(*t*) of the dense sample and the result from the calculation based on the Monomer Attaching/Detaching model, respectively. This figure is prepared by the usage of IGOR Pro 6.34A (https://www.wavemetrics.com/forum/news-and-announcements/igor-634a-now-shipping).

The detailed procedure is summarized in the Supplementary Materials. The best-fit results are shown by the solid lines in Fig. 5. This model successfully reproduced the results of both experiments. We cannot totally exclude the possibility of dimer as exchanging units, hence we also calculated the *I*_0_(*t*)s based on the dimer Attaching/Detaching model (Fig. S11). In the case of the dilute sample, both of *I*_0_(*t*)s calculated from monomer Attaching/Detaching model and dimer Attaching/Detaching model can reproduce the experimental *I*_0_(*t*). It implies that both models could occur for the dilute sample. On the other hand, *I*_0_(*t*) calculated curve based on monomer Attaching/Detaching model could reproduce the experimental *I*_0_(*t*) of the dense sample but that based on dimer Attaching/Detaching model could not reproduce. This result supports that the main mechanism of subunit exchange in αB-crystallin oligomers is the attaching/detaching of monomer and other small oligomers to/from the αB-crystallin oligomer.

## Summary

Taking advantage of inverse contrast matching small-angle neutron scattering (iCM-SANS), we succeeded in studying the structure of the protein in a dense environment without inter-particle interference. It is expected that this technique is applicable to the structural analysis of proteins under more concentrated systems such as in-cell environments.

It was reported that the quaternary structure of the αB-crystallin oligomer was severely influenced by subtle changes in the external environment. It is considered that the quaternary structure of the αB-crystallin oligomer is regulated by subunit exchange between αB-crystallin oligomers. To unveil the unsettled mechanism of its subunit exchange, we studied it at two different protein concentrations: 28.5 mg/mL (dense sample) and 0.45 mg/mL (dilute sample) through iCM-SANS. Interestingly, the exchange rate of the dense sample was the same as that of the dilute sample. It was considered that revealing the distribution of the association number of αB-crystallin should offer a clue for an appropriate model. Analytical ultracentrifugation (AUC) measurements were then performed on both dense and dilute αB-crystallin solutions. Regardless of the protein concentration, it revealed the coexistence of oligomers and small molecular weight components, which were dominated by monomers. We then introduced the event of attaching and detaching monomers and other small oligomers to/from oligomers into the model. This model successfully reproduced the experimental results for both samples. It was concluded that the attaching/detaching of monomers and other small oligomers regulates the subunit exchange in αB-crystallin oligomers. Although further experimental validation is needed, it is also expected that this subunit exchange is related to the function of αB-crystallin chaperone activity.

## Materials and methods

### Preparation of human recombinant hydrogenated and partially deuterated αB-crystallin oligomers

The detailed procedure preparation of hydrogenated (h-) αB-crystallin has already been described in our previous work^13^. For the preparation of partially deuterated (*p*d-) αB-crystallin, *E. coli* transformants were firstly pre-cultured in a 5.0 mL Luria–Bertani (LB) culture solution dissolved in approximately 30% D_2_O containing 50.0 μg/mL ampicillin (Amp) for overnight at 37 °C. As a next step, 50.0 μL of such pre-cultured solution was subsequently added to 5.0 mL LB medium dissolved in approximately 60% D_2_O containing 50.0 μg/mL Amp and further pre-cultured for overnight at 37 °C. Then, the cells were collected by centrifugation and resuspended in M9 minimal media containing deuterated glucose (1.5 g/L), hydrogenated glucose (0.5 g/L), autoclaved milliQ (250.0 mL), and 99.8% D_2_O (750.0 mL). The cells were cultured for approximately 12 h (OD_600_ = 0.6) at 37 °C, after which the expression of αB-crystallin was induced by isopropyl-1-thio *β*-d-galactopyranoside at the final concentration of 0.3 mM, and the cells were grown for an additional 10–12 h at 37 °C. The detailed procedures for the purification of αB-crystallin have already been reported in our previous work^13^ as well. Prior to SANS measurements, both *p*d- and h-αB-crystallin solutions were dialyzed against full D_2_O buffer (99.6% D_2_O ratio) to exchange the exchangeable H atoms in αB-crystallin to D atoms.

### MALDI-TOF mass spectrometry

Saturated solutions of sinapinic acid in TA30 (30% acetonitrile in 70% of 0.1% TFA in water) solvent and h-αB-crystallin (or *p*d-αB-crystallin) were mixed with the volume ratio of 9 to1. Such prepared solutions were dropped on the MALDI plate and let the crystallization after drying it. The measurement was performed with MALDI-TOF Mass Spectrometry^20^ (microflexLT: Bruker Daltonics) under the positive ion mode. Mass spectra data were recorded with flexControl and analyzed with FlexAnalysis (Bruker Daltonics).

### Determination of degree of deuteration of *p*d-αB-crystallin

To determine the degree of deuteration of *p*d-αB-crystallin, we performed MALDI-TOF mass spectrometry measurements on both *p*d- and h- αB-crystallin in H_2_O buffer (Fig. S12). Referring to the sequence of amino acid residue of αB-crystallin, the number of non-exchangeable H atoms^21^ in αB-crystallin was calculated to 1098. Then, the degree of deuteration of *p*d-αB-crystallin was calculated to 70.4% from the difference of *m*/*z* value (= 773.2) between two samples.

### Fourier transform infrared (FT-IR) spectroscopy

FT-IR spectroscopy^22^ was performed with FT/IR-4600 spectrophotometer (JASCO, Tokyo, Japan) equipped with a triglycine sulfate detector and Ge/KBr beam splitter and ATR PRO ONE. Spectra were recorded in the wavenumber covering from 500 to 2000 cm^−1^ with the resolution of 4 cm^−1^.

### Determination of D_2_O ratio

Utilizing the difference of frequency of bending vibration of H–O–D bonding and that of D–O–D bonding^23^, Fourier transform infrared (FT-IR) spectroscopy was performed in order to determine the D_2_O ratio of buffer. The peak at around 1200 cm^−1^ corresponds to the frequency of bending vibration of D–O–D bonding (Fig. S13), then the integrated intensity from 1150 to 1250 cm^−1^ was calculated by changing the volume fraction of D_2_O. From the integrated intensity as a function of volume fraction of D_2_O, the D_2_O ratio of our prepared D_2_O buffer was calculated to 99.6%.

We further confirmed the D_2_O ratio in the buffer by checking the *I*_0_ value OLG(0) at the concentration of 0.45 mg/mL in this full D_2_O buffer (99.6% D_2_O ratio) and *I*_0_ was calculated to 0.24 ± 0.01 cm^−1^ from the Guinier analysis (Fig. S14). Considering the exchange of H atoms to D atoms in OLG(0) in this buffer (99.6% D_2_O ratio), the expected *I*_0_ value was computed to 0.24 cm^−1^, which is quite consistent with the experimental one.

Namely, the D_2_O ratio in the buffer was confirmed to 99.6% from both calculation and FT-IR measurement.

### Small-angle neutron scattering (SANS)

SANS measurements were performed using QUOKKA installed at the Australian Nuclear Science and Technology Organization (ANSTO, Lucas Heights, NSW, Australia). The wavelength, wavelength distribution, the source to sample distance, the sample to detector distance, guides, source aperture diameter and guard aperture diameter were set to 6 Å, 10%, 6 m, 6 m, guide 6 (g6), 50 mm and 12.5 mm, respectively. With this configuration, the *Q* range covered from 0.012 to 0.1 Å^−1^. Further detailed instrumental information should be referred to instrumental paper of QUOKKA^24^. The cylindrical cell with the thickness of 1 mm was utilized for the measurement of dilute, dense samples and 100% D_2_O buffer. The transmission measurements were performed before and after the time-resolved SANS measurements at the exposure time of 120 s. The exposure time for one snapshot in the time course measurement was set to 600 s with a time interval of 1200 s. The measurement procedure for both dense and dilute samples is shown in Fig. S15. The average total counts at detector reached 1.1 × 10^6^ for both samples under this exposure time, meaning that error is less than 0.1%. After the correction for detector efficiency and masking of bad pixels, the obtained two-dimensional scattering patterns were converted to one-dimensional scattering profiles. Subsequently, such obtained one-dimensional scattering profiles were converted to absolute intensity using direct beam method. The scattering profiles from empty cell (*I*(*Q*)_cell_), cadmium (*I*(*Q*)_dark_), solvent (*I*(*Q*)_solv_), sample (*I*(*Q*)_samp_) were used for the data reduction. Utilizing following equations, *I*(*Q*) from solute (*I*(*Q*)_solute_) was calculated.

$$\begin{aligned} I\left( Q \right)_{{{\text{cellc}}}} & = I\left( Q \right)_{{{\text{cell}}}} /T_{{{\text{cell}}}} - I\left( Q \right)_{{{\text{dark}}}} \\ I\left( Q \right)_{{{\text{solvc}}}} & = I\left( Q \right)_{{{\text{solv}}}} /T_{{{\text{solv}}}} - I\left( Q \right)_{{{\text{dark}}}} - I\left( Q \right)_{{{\text{cellc}}}} \\ I\left( Q \right)_{{{\text{solute}}}} & = I\left( Q \right)_{{{\text{samp}}}} /T_{{{\text{samp}}}} - I\left( Q \right)_{{{\text{cellc}}}} - I\left( Q \right)_{{{\text{dark}}}} - \left( {{1} - cv} \right)I\left( Q \right)_{{{\text{solvc}}}} , \\ \end{aligned}$$

where *I*(*Q*)_cellc_, *I*(*Q*)_solvc_, *T*_cell_, *T*_solv_, *T*_samp_, *c* and *v* correspond to the corrected *I*(*Q*) from cell, the corrected *I*(*Q*) from solvent, the transmission of empty cell, the transmission of solvent, the transmission of sample, concentration and the specific volume calculated from the sequence of amino acid residue, respectively. *I*(*Q*)_solute_s from the dilute and dense samples were utilized for further data analysis. The data reductions up to obtaining *I(Q)*_*solute*_ were performed with the macro of IGOR Pro from NIST^25^, which is adapted with instrumental parameters of QUOKKA.

### Analytical ultracentrifugation (AUC)

The AUC experiments were performed with XL-I (Beckmann Colter). For the observation of both oligomers and small molecular weight components, the measurements were performed with Rayleigh interference optics at 40,000 rpm at 37 °C. To focus on the observation of small molecular weight components in more detail, the measurements were also performed with Rayleigh interference optics at 60,000 rpm at 37 °C. The distribution of the sedimentation coefficient (*c*(*s*_20,w_)) was obtained from the analysis with SEDFIT^26^.

### Dynamic light scattering (DLS)

DLS measurements were performed with a system equipped with a 22-mW He–Ne laser, an avalanche photodiode (APD, ALV, Germany) mounted on a static/dynamic compact goniometer, ALV/LSE-5003 electronics, and an ALV-5000 Correlator (ALV-Laser Vertriebsgesellschaft GmbH, Langen, Germany). The measurements were performed at 37 °C. CONTIN analysis^27^ was applied to obtain the distribution of the decay rate.

## Supplementary Information

Supplementary Information

## Data Availability

The datasets generated and analyzed during the current study are available from the corresponding authors upon reasonable request.
