# Supplementary material for: Elucidation of the mechanism of subunit exchange in αB crystallin oligomers
Source: Sci Rep. 2021 Jan 28;11:2555. doi: 10.1038/s41598-021-82250-z (PMC7843597; doi:10.1038/s41598-021-82250-z)
Supplement: Supplementary file 1 — Supplementary Information [file 41598_2021_82250_MOESM1_ESM.pdf]

Supplementary Materials

Elucidation of the mechanism of subunit exchange in αB crystallin oligomers

Rintaro Inoue[1*], Yusuke Sakamaki[1], Takumi Takata[1], Kathleen Wood[2], Ken Morishima[1], Nobuhiro Sato[1], Aya Okuda[1], Masahiro Shimizu[1], Reiko Urade[1], Noriko Fujii[1] and Masaaki Sugiyama[1*]

[1]*Institute for Integrated Radiation and Nuclear Science, Kyoto University, Kumatori, Sennan-gun, Osaka 590-0494 JAPAN.*

[2]*Australian Nuclear Science and Technology Organization, Lucas Heights, NSW Australia.*

*Corresponding authors. R. I. rintaro@rri.kyoto-u.ac.jp, M. Sugiyama sugiyama@rri.kyoto-u.ac.jp

This PDF file includes:

Fig. S1-15 and the detailed explanation for the evaluation of exchangeable subunit number in oligomer, collision model and monomer attaching/detaching model.

The scattering profiles of OLG(26) at the concentration of 0.45 mg/mL in full D_2O buffer

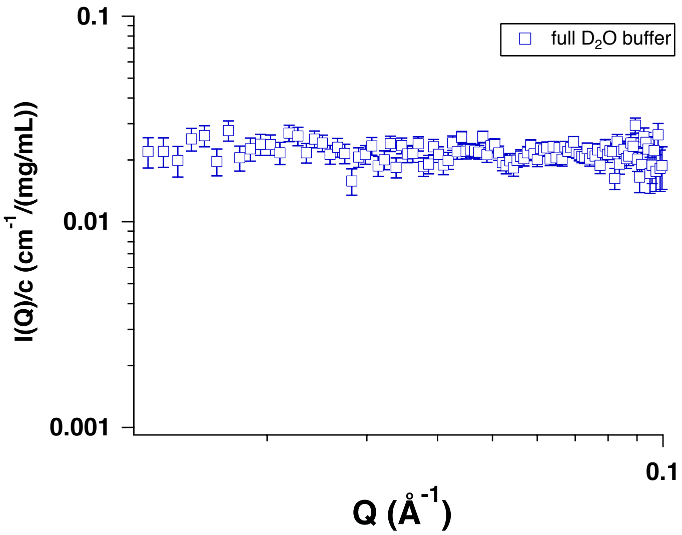

Fig. S1. The SANS profile from dilute OLG(26) at the concentration of 0.45 mg/mL in full D_2O buffer (99.6% D_2O ratio) (blue square), respectively.

This figure is prepared by the usage of IGOR Pro 6.34A (https://www.wavemetrics.com/forum/news-and-announcements/igor-634a-now-shipping).

The scattering visibilities for OLG(26) at 28.5 mg/mL and OLG(0) at 0.45 mg/mL

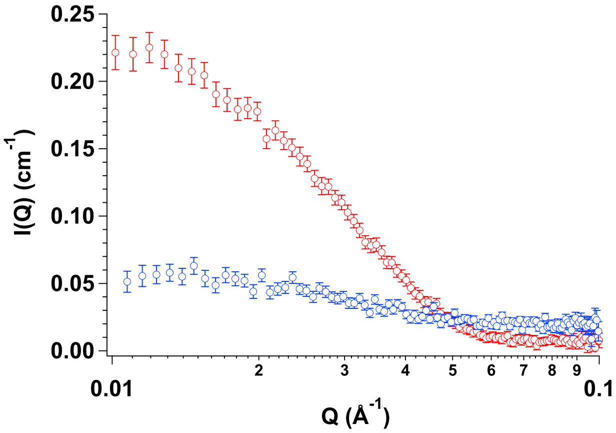

Fig. S2. SANS profiles from OLG(26) at 28.5 mg/mL (blue circle) and OLG(0) at 0.45 mg/mL (red circle) in full D_2O buffer (99.6% D_2O ratio).

This figure is prepared by the usage of IGOR Pro 6.34A (https://www.wavemetrics.com/forum/news-and-announcements/igor-634a-now-shipping).

Gunier plots of dilute and dense samples

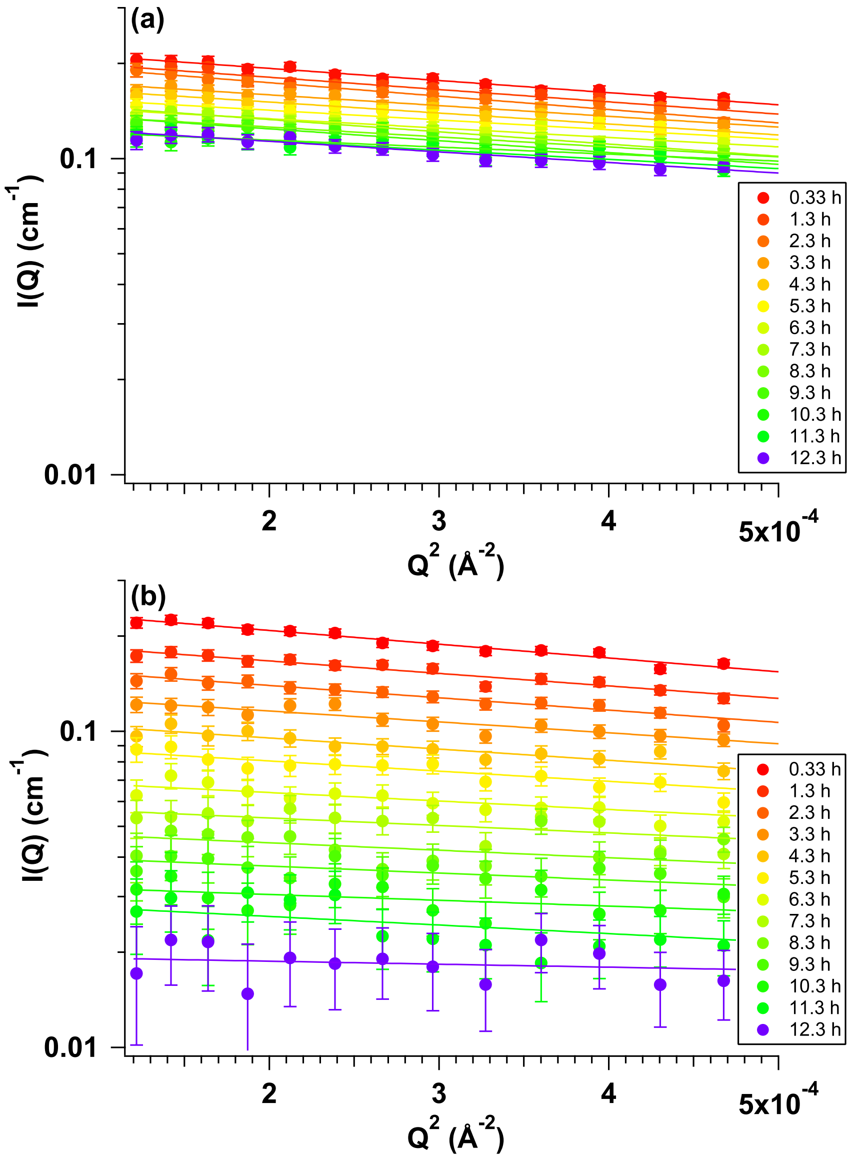

Fig. S3. Guinier plot of (a) dilute and (b) dense samples after mixing with OLG(0) and OLG(26). Red to purple circle and solid lines correspond to the SANS profile and results of fit with Guinier function at 0.33 h and to that at 12.3 h.

This figure is prepared by the usage of IGOR Pro 6.34A (https://www.wavemetrics.com/forum/news-and-announcements/igor-634a-now-shipping).

Time dependence of R_g from the dilute and dense samples

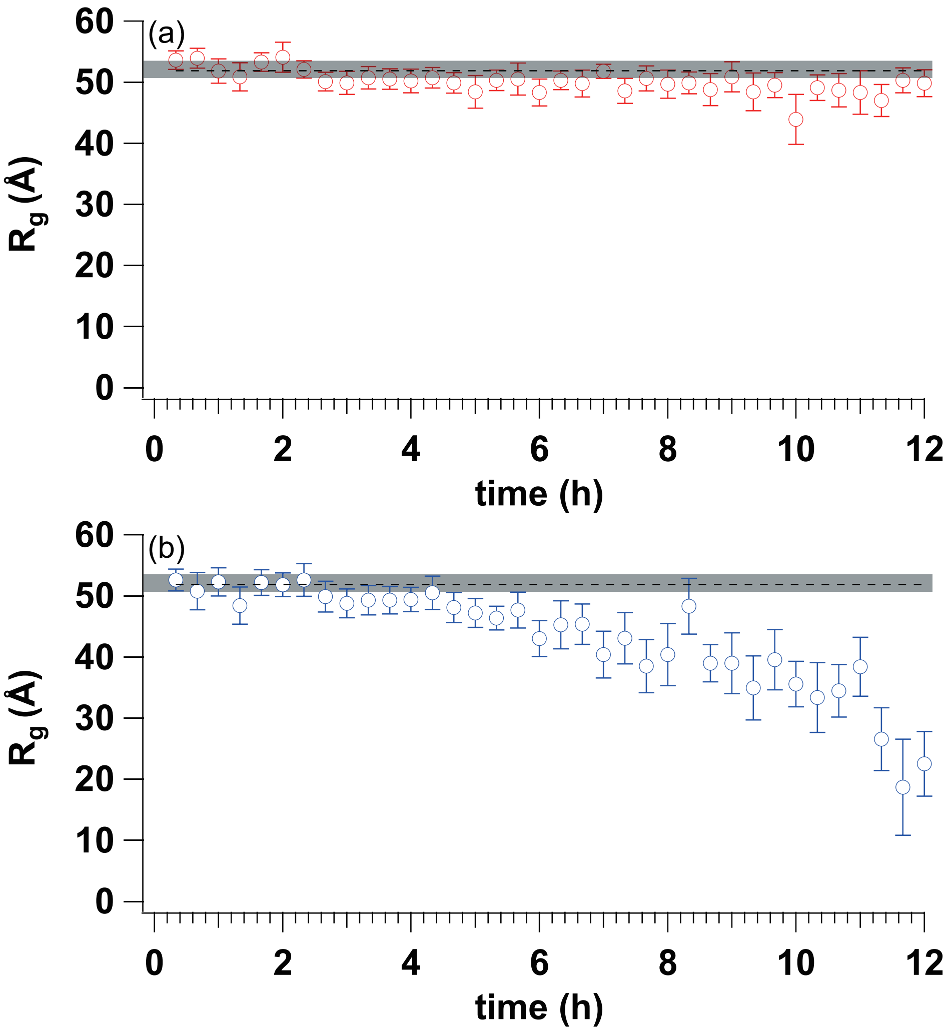

Fig. S4. Time dependence of R_g from (a) dilute (red circle) and (b) dense (blue circle) samples. The black dashed lines and gray shaded areas correspond to R_g of OLG(0) at the concentration of 0.45 mg/mL determined by SAXS measurements and its error bar, respectively.

This figure is prepared by the usage of IGOR Pro 6.34A (https://www.wavemetrics.com/forum/news-and-announcements/igor-634a-now-shipping).

Exchangeable subunit number in oligomer

We adopted the average association number of αB-crystallin oligomers as 26. Here, we estimated the number of exchangeable subunits n in the αB-crystallin oligomer with an association number of 26. The A value in eq. (2), which is the ratio of intensity at the equilibrium state to the initial intensity, is a function of n, and then $A(n)$ is expressed by the following equation:

$$A(n)=\frac{\sum_{m=0}^{n}\left({}_nC_m z^{(n+1-m)}m^2+{}_nC_m z^m(26-m)^2\right)}{\sum_{m=0}^{n}\left({}_nC_m z^{(n+1-m)}+{}_nC_m z^m\right)}\frac{(z+1)}{26^2}, \qquad \text{Eq. (S1)}$$

where m and z correspond to the number of *p*d-subunits in an oligomer and the ratio of the initial protein concentration of OLG(26) to OLG(0), respectively. $A(n)$ is plotted in Fig. S5. Considering the error bars, the experimentally determined A values coincided with $A(26)$ for both the dilute and dense samples. This means that all subunits in the αB-crystallin oligomer with an association number of 26 were exchanged regardless of protein concentration.

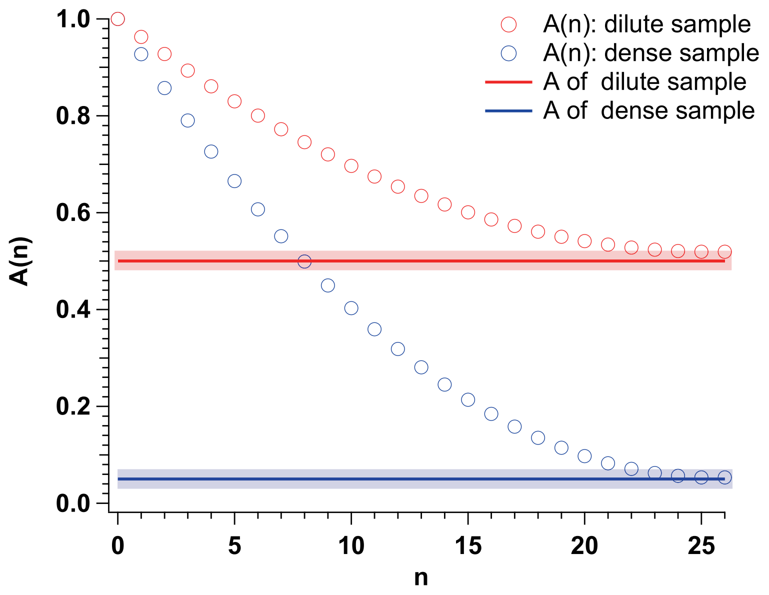

Fig. S5. $A(n)$ values and experimentally determined A values for the dilute and dense samples. The red and blue circles correspond to the calculated $A(n)$ values for the dilute and dense samples, respectively. The red and blue solid lines indicate the experimentally determined A values for the dilute and dense samples, respectively, and the pink and blue shaded zones show the error bars of experimentally determined A values for the dilute and dense samples, respectively.

This figure is prepared by the usage of IGOR Pro 6.34A (https://www.wavemetrics.com/forum/news-and-announcements/igor-634a-now-shipping).

Collision model

We assumed that subunit exchange could occur due to a random collision between two αB-crystallin oligomers. It is also assumed that the αB-crystallin oligomers always exchange their subunits when the two oligomers collide. Namely, the collision frequency calculated from this model is comparable to the exchange rate. $I_0(t)$ was calculated using the following procedure.

1. We set the parameters and a function as follows:
   - Collision frequency between two oligomers: *k*. (k_l at dilute sample, k_h at dense sample)
   - Calculation step: *cs*
   - Number of *p*d-subunits in an oligomer: *m*
   - Number of αB-crystallin oligomers with *m* (OLG(*m*)) at *cs*: $N[m](cs)$
   - Forward scattering intensity at *cs*: $I_0(cs)$
   - Normalized forward scattering intensity at *cs*: $I_{0,\,nor}(cs) = I_0(cs)/I_0(0)$

2. Supposing the collision between OLG(*i*) and OLG(*j*) at *cs*, the numbers of six oligomers, OLG(*i*), OLG(*i*-1), OLG(*i*+1), OLG(*j*), OLG(*j*-1), and OLG(*j*+1), will be affected at *cs*+1. We should also consider that there are two cases of subunit exchange. One is that the *p*d-subunit in OLG(*i*) exchanges the h-subunit in OLG(*j*) (Case 2-1). Because both the number of *p*d-subunits in OLG(*i*) and that of the h-subunit in OLG(*j*) is proportional to the collision probability, the collision probability is given by (*i*/26) * ((26 - *j*)/26) in Case 2-1. The other is that the h-subunit in OLG(*i*) exchanges with the *p*d-subunit in OLG(*j*) (Case 2-2). In Case 2-2, the collision probability is given by (26-*i*)/26) * (*j*/26). Depending on the cases (Case 2-1 and Case 2-2), the following equations as a function of *cs* can be written.

Case 2-1

$N[i](cs+1) = N[i](cs) - kN[i](cs)N[j](cs) * (i/26) * ((26-j)/26),$

$N[i-1](cs+1)=N[i-1](cs) + kN[i](cs)N[j](cs) * (i/26) * ((26 - j)/26),$

$N[j](cs+1) = N[j](cs) - kN[i](cs)N[j](cs) * (i/26) * ((26 - j)/26),$

$N[j+1](cs + 1) = N[j+ 1](cs) + kN[i](cs)N[j](cs) * (i/26) * ((26-j)/26),$

Eq. (S2)

Case 2-2

$N[i](cs+1) = N[i](cs) - kN[i](cs)N[j](cs) * ((26 - i)/26) * (j/26)$,

$N[i+1](cs +1) = N[i +1](cs) + kN[i](cs)N[j](cs) * ((26 - i)/26) * (j/26)$,

$N[j](cs+1) = N[j](cs) - kN[i](cs)N[j](cs) * ((26 - i)/26) * (j/26)$,

$N[j-1](cs+1) = N[j-1](cs) - kN[i](cs)N[j](cs) * ((26 - i)/26) * (j/26)$,

Eq. (S3)

3. For the dilute sample, we set the initial values as follows: $N[0](0) = 1.0$, $N[26](0) = 1.0$, and $k_1 = 0.01$. Then, $N[m](cs)$ ($0 \leq m \leq 26$) is calculated with the successive substitution method using Eqs. (S2) and (S3).

4. $I_0(cs)$ and $I_{0,\text{ nor}}(cs)$ are given by the following equations, respectively :

$$I_0(cs) = \sum_{m=0}^{26} N[m](cs)(26 - m)^2 , \quad \text{Eq. (S4)}$$

$$I_{0,\text{ nor}}(cs) = \frac{I_0(cs)}{I_0(0)} , \quad \text{Eq. (S5)}$$

5. $I_{0,\text{ nor}}(cs)$ from the dilute sample exhibits decay equation as a function of cs. To convert $I_{0,\text{ nor}}(cs)$ to the experimentally derived $I_0(t)$ of the dilute sample, the time scaling factor (sf) was calculated. The results are plotted in Fig. S6 (a).

6. For the dense sample, we set the initial values as follows: $N[0](0) = 1.0$ and $N[26](0) = 63.0$. Then, $I_{0,\,nor}(cs)$ of the dense sample was calculated by varying k. Utilizing the determined sf at procedure 5, k (k_h) that can reproduce $I_0(t)$ of the dense sample was estimated. The results are plotted in Fig. S6 (b).

7. Finally, the collision ratio for the dense sample to the dilute sample (= k_h/k_d) was calculated to be 31.5.

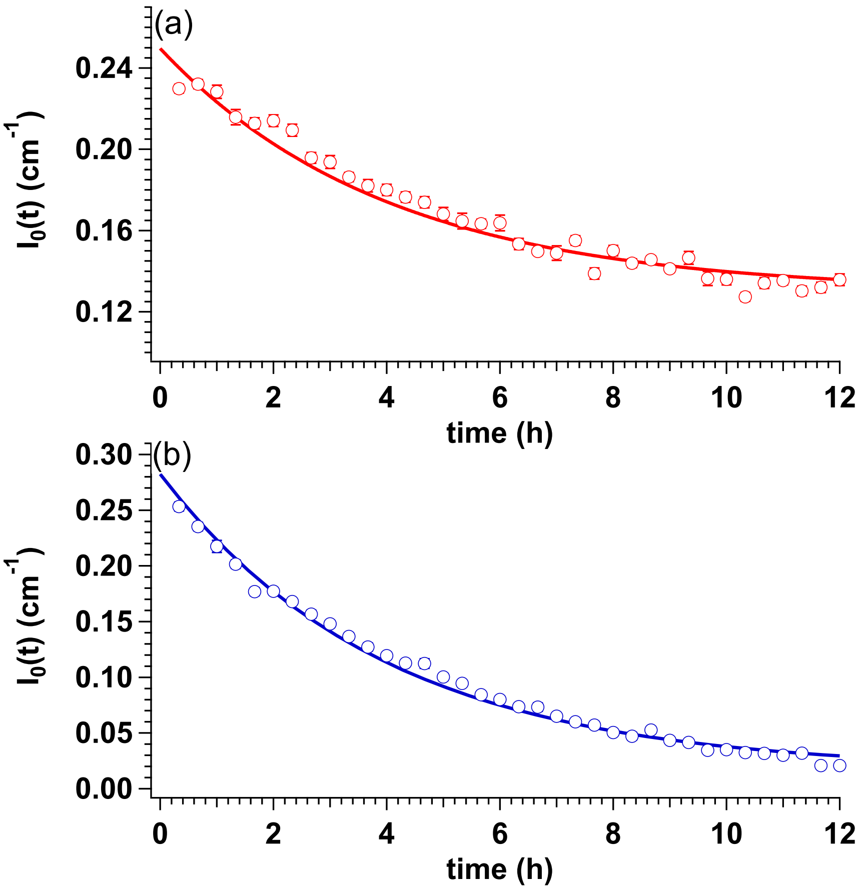

Fig. S6. $I_0(t)$s of the dilute and the dense samples, and the calculated curves based on the collision model. (a) The red circle and the red solid curve correspond to $I_0(t)$ of the dilute sample and the result of the calculation based on the collision model, respectively. (b) The blue circle and the blue solid curve correspond to $I_0(t)$ of the dense sample and the result of the calculation based on the collision model, respectively.

This figure is prepared by the usage of IGOR Pro 6.34A (https://www.wavemetrics.com/forum/news-and-announcements/igor-634a-now-shipping).

8. It is considered that the collision frequency is dependent on both the diffusion constant and the concentration of particles. We can calculate the collision frequency from another experimental approach for the examination of the collision frequency rate obtained by iCM-SANS under the collision model. Based on the idea of a diffusion-limited reaction, the collision frequency, k_{ex}, is given by the following equation:

$$k_{ex} = 4\pi N_a N_b (D_a + D_b)(R_a + R_b), \qquad \text{Eq. (S6)}$$

where N_j, D_j, and R_j correspond to the number density, translational diffusion constant, and collision radius of j-particle (j = a, b), respectively. Assuming that the collision radius is equal to the hydrodynamic radii, the k_{ex} values of both samples can be calculated from the results of the dynamic light scattering (DLS) measurements.

9. DLS measurements were performed for αB-crystallin solutions at 0.45 and 28.5 mg/mL at 37°C. Q^2 dependencies of the relaxation rates (Γ) are shown in Figure S7. The translational diffusion coefficients (D_t) were calculated from the relationship $\Gamma = D_t Q^2$. D_ts of αB-crystallin at 0.45 and 28.5 mg/mL were 3.70 ± 0.50)×10^{-7} cm^2/s and (3.51 ± 0.30) × 10^{-7} cm^2/s, respectively, and the corresponding hydrodynamic radii were calculated using the Einstein–Stokes equation.

10. The k_{ex} values of the dense and dilute samples were calculated to be (3.49 ± 0.04) × 10^8 $s^{-1}m^{-3}$ and (5.51 ± 0.08) × 10^6 $s^{-1}m^{-3}$, respectively. The ratio of the collision frequency of dense sample to dilute sample by this approach was calculated to be 63.3, which critically deviates from that of the scattering experiment under the collision model (31.5). It is concluded that the present model, the collision model, is excluded as an appropriate model to explain the subunit exchange mechanism.

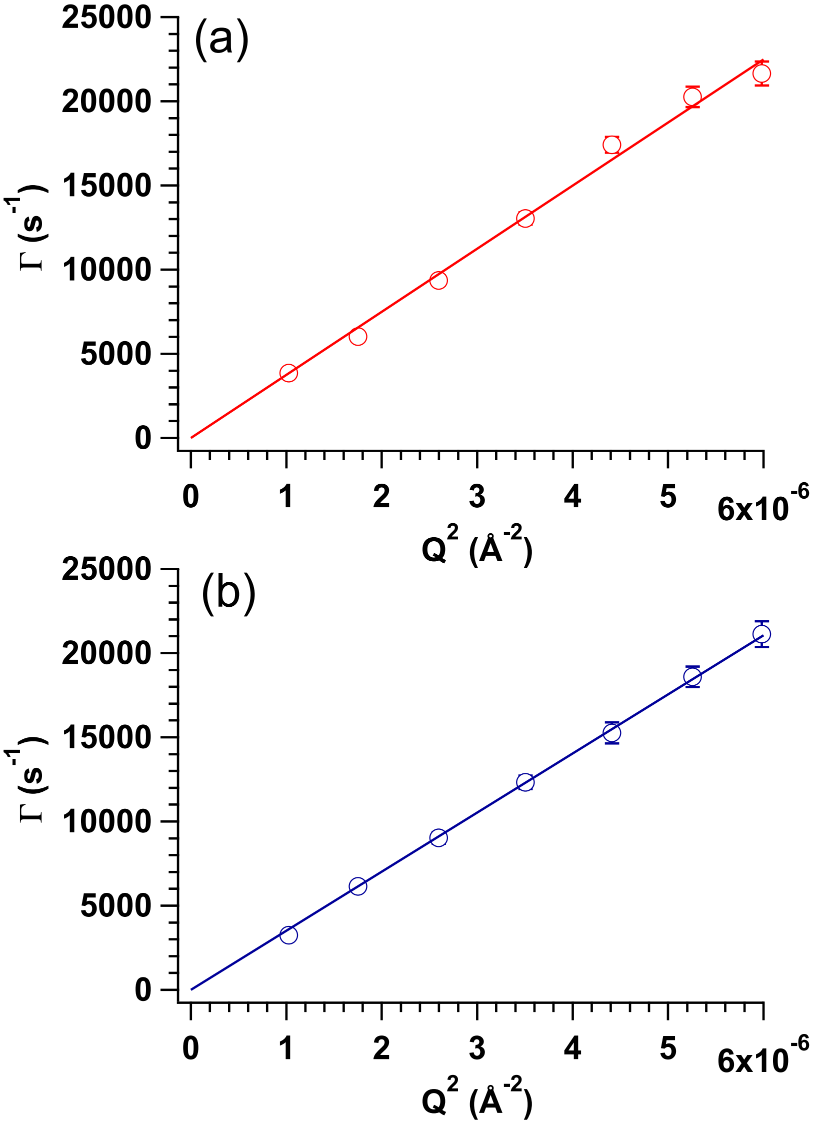

Fig. S7. Q^2 dependence of Γs from αB-crystallin solution at (a) 0.45 and (b) 28.5 mg/mL at 37°C. The red and blue solid lines correspond to the fits with $\Gamma = D_t Q^2$.

This figure is prepared by the usage of IGOR Pro 6.34A (https://www.wavemetrics.com/forum/news-and-announcements/igor-634a-now-shipping).

Sedimentation velocity analysis of dilute and dense samples measured at 60000 rpm

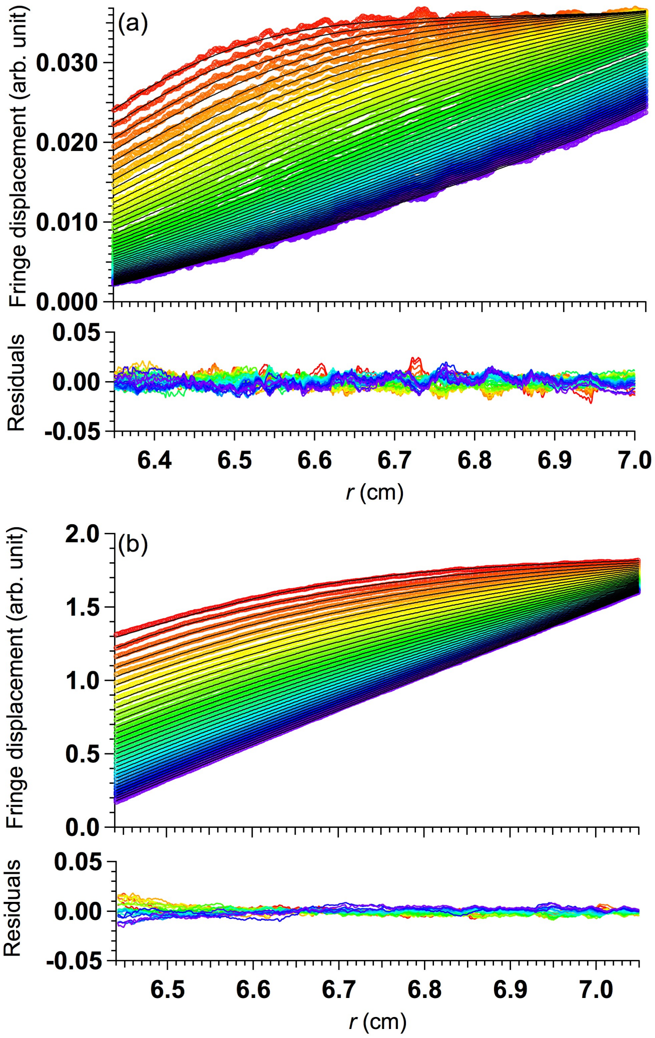

Fig. S8. Sedimentation velocity analysis of dilute and dense samples. (a) Interference data of the dilute sample, sedimenting at 60000 rpm in 12 mm centerpieces, are shown with color temperature gradient indicating the boundary migrating from left to right. The bset-fit curves are show by solid lines and the lower panel corresponds to the residuals of the fit. (b) Interference data of the dense sample, sedimenting at 60000 rpm in 3 mm centerpieces, are shown with color temperature gradient indicating the boundary migrating from left to right. The bset-fit curves are show by solid lines and the lower panel corresponds to the residuals of the fit.

This figure is prepared by the usage of IGOR Pro 6.34A (https://www.wavemetrics.com/forum/news-and-announcements/igor-634a-now-shipping).

Relationship between $s_{20,w}$ and M_w

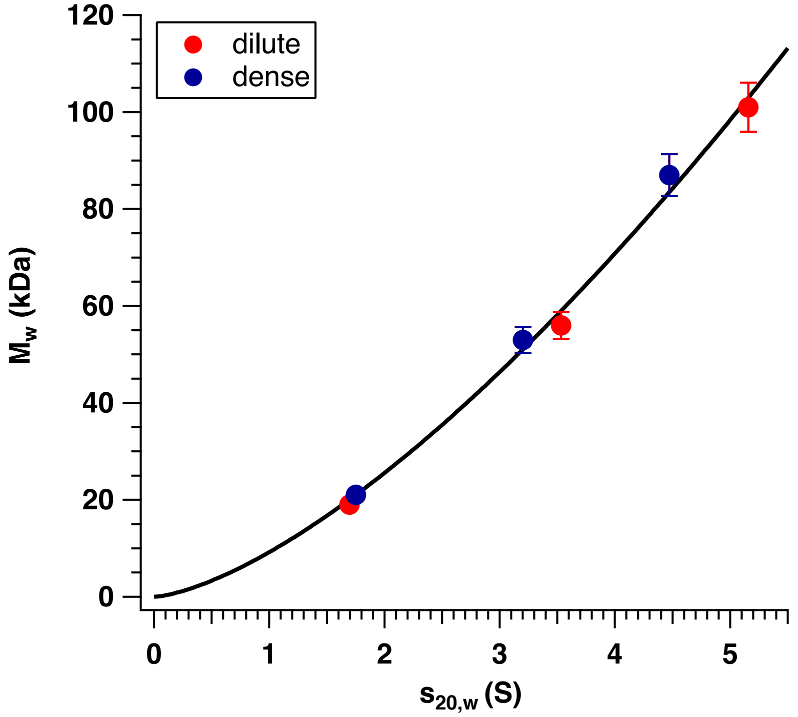

Fig. S9. M_w as a function of $s_{20,w}$ and black solid line corresponds to the relationship of $M_w \sim s_{20,w}^{1.5}$.

This figure is prepared by the usage of IGOR Pro 6.34A (https://www.wavemetrics.com/forum/news-and-announcements/igor-634a-now-shipping).

Monomer Attaching/Detaching model

It is considered that attaching/detaching monomers contribute to the subunit exchange in αB-crystallin oligomers (refer to Fig. 5). $I_0(t)$ was calculated using the following procedure.

1. We defined the rates of attaching and detaching monomers as k_a and k_d, respectively.

2. In order to consider the distribution of the association number in αB-crystallin oligomers in the model system, we referred to the AUC results.

2.1 The number of oligomers with an association number of *na* is defined as *N*[*na*].

2.2 We set n_1 and n_2, which corresponded to the lower (= 20) and upper limit (= 32) of the association number of the αB-crystallin oligomer, respectively. To simplify the calculation, only the monomer was taken into consideration for the small molecular weight components.

2.3 Then, we set *N*[1] = 0.12, *N*[20] = 0.18, *N*[21] = 0.31, *N*[22] = 0.47, *N*[23] = 0.65, *N*[24] = 0.83, *N*[25] = 0.95, *N*[26] = 1, *N*[27] = 0.95, *N*[28] = 0.83, *N*[29] = 0.65, *N*[30] = 0.47, *N*[31] = 0.31 and *N*[32] = 0.18, respectively. The distribution of monomers and αB-crystallin oligomers is plotted in Fig. S10.

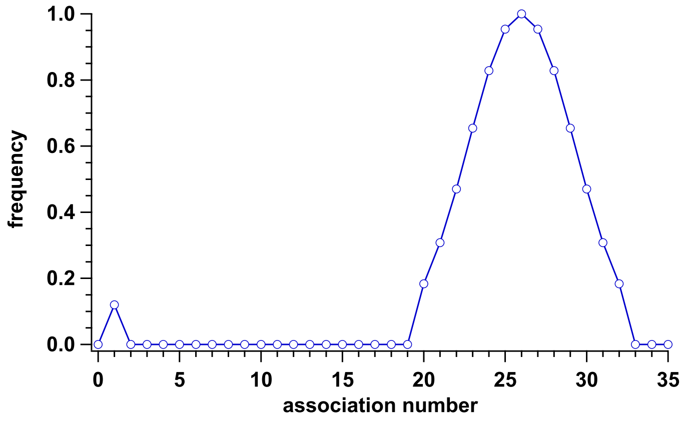

Fig. S10. Distribution of the association number of αB-crystallin oligomers used in the Monomer Attaching/Detaching model.

This figure is prepared by the usage of IGOR Pro 6.34A (https://www.wavemetrics.com/forum/news-and-announcements/igor-634a-now-shipping).

3. It is considered that the weight ratio of small molecular components to oligomer can be used to determine the ratio of k_a to k_d. We performed a simple calculation as follows.

3.1 We assumed the coexistence of monomers and oligomers with an association number ranging from 20 (= n_1) to 32 (= n_2), as described in procedure 2.2.

3.2 $N[na](cs)$ corresponds to na at the calculation step: cs

3.3 To describe $N[na]$ at $cs + 1$ ($N[na](cs + 1)$), the following equations as a function of cs can be written depending on the cases (Cases 3-1, 3-2, 3-3, and 3-4).

Case 3-1: $na = n_2$

$$N[na](cs+1) = N[na](cs) - k_d N[na](cs) + k_a N[na-1](cs)\,N[1](cs), \tag{S7}$$

Case 3-2: $n_1+1 \le na \le n_2-1$

$$N[na](cs+1) = N[na](cs) + k_d N[na+1](cs) - k_a N[na](cs)N[1](cs) - k_d N[na](cs). + k_a N[na-1](cs)\,N[1](cs), \tag{S8}$$

Case 3-3: $na = n_1$

$$N[na](cs+1) = N[na](cs) + k_d N[na+1](cs) - k_a N[na](cs)\,N[1](cs), \tag{S9}$$

Case 3-4: $na=1$

$$N[na](cs+1) = N[na](cs) + \sum_{i=n_1+1}^{n_2} k_d N[i](cs) - \sum_{j=n_1}^{n_2-1} k_a N[j](cs)N[na](cs), \tag{S10}$$

3.4 $N[na](0)$ ($na = 1$, $n_1 \leq na \leq n_2$) was obtained from the number distribution described in procedure 2.3. By changing the ratio of k_a to k_d, $N[na]$(cs) was calculated with the successive substitution method using Eqs. (S7), (S8), (S9), and (S10).

3.5 The ratio of monomers to total oligomers with the association number of na ($n_1 \leq na \leq n_2$) ($r(cs)$) was calculated for each cs.

$$r(cs) = \frac{N[1](cs)}{\sum_{na=n_1}^{n_2} N[na](cs)},$$

Eq. (S11)

3.6 To fix the ratio for k_a to k_d, we must consider the condition that $r(cs)$ is equal to 0.015 regardless of cs. The ratio of k_a to k_d was determined to 8.6 for both the dilute and the dense samples. This ratio was then utilized for the later calculation.

4. We set the parameters and a function as follows:

- The rates of attaching and detaching monomers as k_a and k_d
- Calculation step: cs
- Association number in αB-crystallin: na ($na = 1$, $n_1 \leq na \leq n_2$)
- Number of pd-subunits in an oligomer: i ($0 \leq i \leq na$)
- Number of αB-crystallin with na and i at cs: $N_{na}[i](cs)$
- Forward scattering intensity at cs: $I_0(cs)$.
- Normalized forward scattering intensity at cs: $I_{0,\text{nor}}(cs) = I_0(cs)/I_0(0)$

5. Depending on the association number in αB-crystallin, three cases should be taken into consideration (Cases 5-1, 5-2, and 5-3). We then explain each case step by step.

The first case (Case 5-1) is that the association number of αB-crystallin is equal to n_2. There exist two cases that influence i in $N_{na}[i](cs+1)$. First, i of $N_{na}[i](cs+1)$ is equal to that of $N_{na}[i](cs)$ at $cs+1$ ($N_{na}[i](cs+$

1)). The other is the increment of i at cs+1 ($N_{na}[i + 1](cs+1)$). It should be noted that the detaching probability of the pd-subunit in $N_{na}[i]$ is given by i/na. Both $N_1[1](cs+1)$ and $N_1[0](cs+1)$ are affected by the detachment of the pd- (or h-) subunit from N_{na}[i](cs). The following equations can be written.

Case 5-1: $\underline{na}=n_2$

$N_{\underline{na}}[i](cs+1)=N_{\underline{na}}[i](cs)-k_d*N_{\underline{na}}[i](cs)+k_a*N_{\underline{na}-1}[i](cs)*N_1[0](cs)$,

$N_{\underline{na}}[i+1](cs+1)= N_{\underline{na}}[i+1](cs)+k_a*N_{\underline{na}-1}[i](cs)*N_1[1](cs)$,

$N_1[1](cs+1)=N_1[1](cs)+k_d*N_{\underline{na}}[i](cs)*i/\underline{na}$,

$N_1[0](cs+1)= N_1[0](cs)+k_d*N_{\underline{na}}[i](cs)*(\underline{na}-i)/\underline{na}$,

Eq. (S12)

As a next step, we consider Case 5-2 in which the association number in αB-crystallin ranges from n_1+1 to n_2-1. There exist three cases that influence i in $N_{na}[i](cs+1)$. First, i of $N_{na}[i](cs+1)$ is equal to that of $N_{na}[i](cs)$ at cs+1 ($N_{na}[i](cs+1)$). The second is the decrement of i at cs+1 ($N_{na}[i-1](cs+1)$). The third is the increment of i at cs+1 ($N_{na}[i + 1](cs+1)$). As stated above, the detaching probability of the pd-subunit in $N_{na}[i]$ is given by i/na. We also consider the special case for $N_{na}[0](cs+1)$ ($N_{na}[0](cs+1)$). N_1[1](cs +1) and N_1[0](cs+1) are affected by both the detachment of the pd- (or h-) subunit from N_{na}[i](cs) and the attachment of the pd- (or h-) subunit to N_{na}[i](cs). The following equations can be written.

Case 5-2: $n_1+1 \le na \le n_2-1$

$N_{\underline{na}}[i](cs+1)=N_{\underline{na}}[i](cs)-k_d*N_{\underline{na}}[i](cs)- k_a*N_{\underline{na}}[i](cs)*(N_1[0](cs)+ N_1[1](cs))$

$\quad + k_d*N_{\underline{na}+1}[i](cs)*(\underline{na}+1-i)/(\underline{na}+1)+k_a*N_{\underline{na}-1}[i](cs)*N_1[0](cs)$,

$N_{\underline{na}}[i-1](cs+1)= N_{\underline{na}}[i-1](cs)+k_d*N_{\underline{na}+1}[i](cs)*i/(\underline{na}+1)$,

$N_{\underline{na}}[i+1](cs+1)= N_{\underline{na}}[i+1](cs)+k_a*N_{\underline{na}-1}[i](cs)* N_1[1](cs)$,

$N_{\underline{na}}[0](cs+1) = N_{\underline{na}}[0](cs)+k_d*N_{\underline{na}+1}[0](cs)$,

$N_1[1](cs+1)=N_1[1](cs)+k_d*N_{\underline{na}}[i](cs)*i/\underline{na}-k_a*N_{\underline{na}}[i](cs)*N_1[1](cs)$,

$N_1[0](cs+1)= N_1[0](cs)+k_d*N_{\underline{na}}[i](cs)*(\underline{na}-i)/\underline{na}-k_a*N_{\underline{na}}[i](cs)* N_1[0](cs)$,

Eq. (S13)

Finally, we consider Case 5-3 in which the association number in αB-crystallin is equal to n_1. There exist two cases that influence i in $N_{na}[i](cs+1)$. First, i of $N_{na}[i](cs+1)$ is equal to that of $N_{na}[i](cs)$ at $cs+1$ ($N_{na}[i](cs+1)$). The other is the decrement of i at $cs+1$ ($N_{na}[i\text{-}1](cs+1)$). As stated above, the detaching probability of the pd-subunit in $N_{na}[i]$ is given by i/na. We also consider the special case for $N_{na}[0](cs+1)$ ($N_{na}[0](cs+1)$). As a result, $N_1[1]$(cs+1) and $N_1[0]$(cs+1) are affected by the attachment of the pd- (or h-) subunit to N_{na}[i](cs). The following equations can be written.

Case 5-3: $na=n_1$

$N_{\underline{na}}[i](cs+1) = N_{\underline{na}}[i](cs)+k_d*N_{\underline{na}+1}[i](cs)*(na+1-i)/(na+1)$

$\quad -k_a*N_{\underline{na}}[i](cs)*(N_1[0](cs)+ N_1[1](cs)),$

$N_{\underline{na}}[i\text{-}1](cs+1) = N_{\underline{na}}[i\text{-}1](cs)+k_d*N_{\underline{na}+1}[i](cs)*i/(na+1),$

$N_{\underline{na}}[0](cs+1) = N_{\underline{na}}[0](cs)+k_d*N_{\underline{na}+1}[0](cs),$

$N_1[1](cs+1) = N_1[1](cs)- k_a* N_{\underline{na}}[i](cs)* N_1[1](cs),$

$N_1[0](cs+1)=N_1[0](cs)-k_a*N_{\underline{na}}[i](cs)* N_1[0](cs),$

Eq. (S14)

6. $N_{na}[0](0) = N[na]$ ($na = 1$ and $n_1 \le na \le n_2$) (refer to procedure 2.3), $N_{na}[na](0) = N[na]$ ($na = 1$ and $n_1 \le na \le n_2$), and $k_d = 0.0001$ were initially set for the dilute sample. Then, $N_{na}[i](cs)$ can be calculated from the successive substitution method using Eqs. (S12), (S13) and (S14).

7. $I_0(cs)$ and $I_{0,\,\mathrm{nor}}(cs)$ are given by the following equation, respectively:

$$I_0(cs) = \sum_{k=n_1}^{n_2}\sum_{i=0}^{k} N_k[i](cs) * (k-i)^2 + N_1[0](cs) \quad \text{Eq. (S15)}$$

$$I_{0,\,\mathrm{nor}}(cs) = I_0(cs)/I_0(0) \quad \text{Eq. (S16)}$$

8. $I_{0,\,\mathrm{nor}}(cs)$ from the dilute sample exhibits a decay equation as a function of *cs*. To convert the $I_{0,\,\mathrm{nor}}(cs)$ to the experimentally derived $I_0(t)$ of the dilute sample, the time scaling factor (*sf*2) was determined. The final result is shown in Fig. 6 (a).

9. $N_{na}[0](0) = N[na]$ ($na = 1$, $n_1 \leq na \leq n_2$), $N_{na}[na](0) = 63 * N[na]$ ($na = 1$, $n_1 \leq na \leq n_2$), and $k_\mathrm{d} = 0.0001$ were initially set for the dense sample. Then, $I_{0,\,\mathrm{nor}}(cs)$ was calculated for each *cs* as well. Utilizing the determined *sf*2 in procedure 8, the final result is shown in Fig. 6 (b).

Comparison between monomer Attaching/Detaching and dimer Attaching/Detaching model

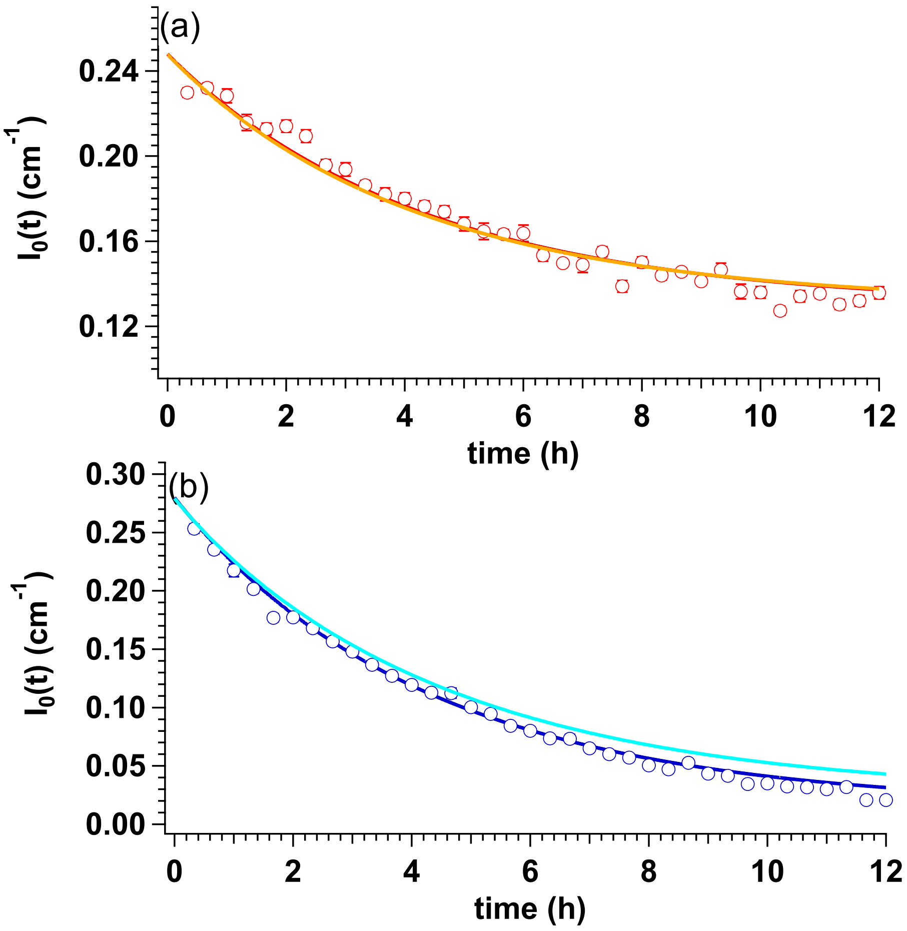

Fig. S11. $I_0(t)$s of the dilute and the dense samples, and the calculated curves based on the monomer Attaching/Detaching and dimer Attaching/Detaching model. (a) The red circle, the red and the yellow solid lines correspond to $I_0(t)$ of the dilute sample, the results of the calculation based on the monomer Attaching/Detaching and the dimer Attaching/Detaching model, respectively. (b) The blue circle, the blue and the light blue solid lines correspond to $I_0(t)$ of the dense sample, the results of the calculation based on the monomer Attaching/Detaching and the dimer Attaching/Detaching model, respectively.

This figure is prepared by the usage of IGOR Pro 6.34A (https://www.wavemetrics.com/forum/news-and-announcements/igor-634a-now-shipping).

MALDI-TOF mass spectra from OLG(0) and OLG(26)

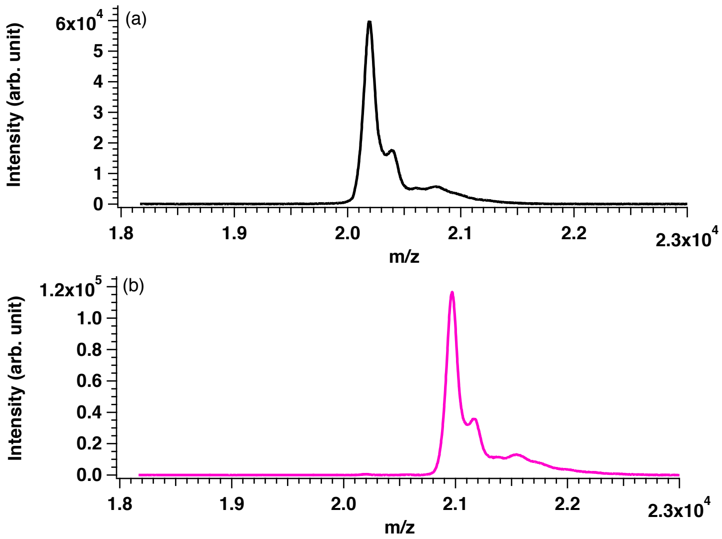

Fig. S12. MALDI-TOF mass spectra from (a) OLG(0) and (b) OLG(26) in H_2O buffer.

This figure is prepared by the usage of IGOR Pro 6.34A (https://www.wavemetrics.com/forum/news-and-announcements/igor-634a-now-shipping).

IR spectra from our prepared D_2O buffer, 99.96% D_2O and 90% D_2O.

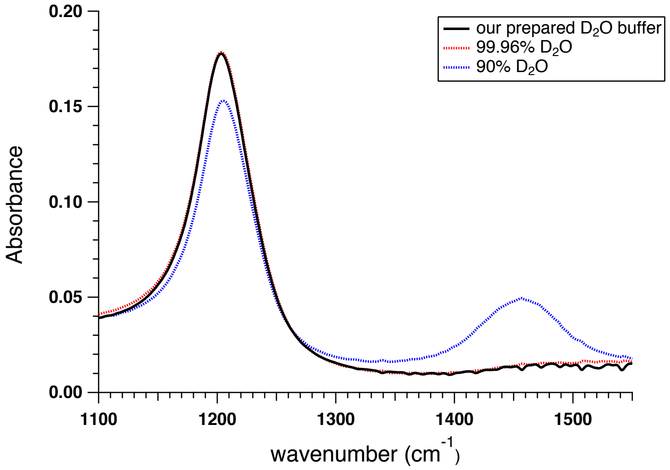

Fig. S13. IR spectra from our prepared D_2O buffer (black solid line), 99.96% D_2O (423459-1PAK from Sigma Aldrich Co.) (red dotted line) and 90% D_2O (blue dotted line), .

This figure is prepared by the usage of IGOR Pro 6.34A (https://www.wavemetrics.com/forum/news-and-announcements/igor-634a-now-shipping).

SANS profile from OLG(0) at the concentration of 0.45 mg/mL in full D_2O buffer

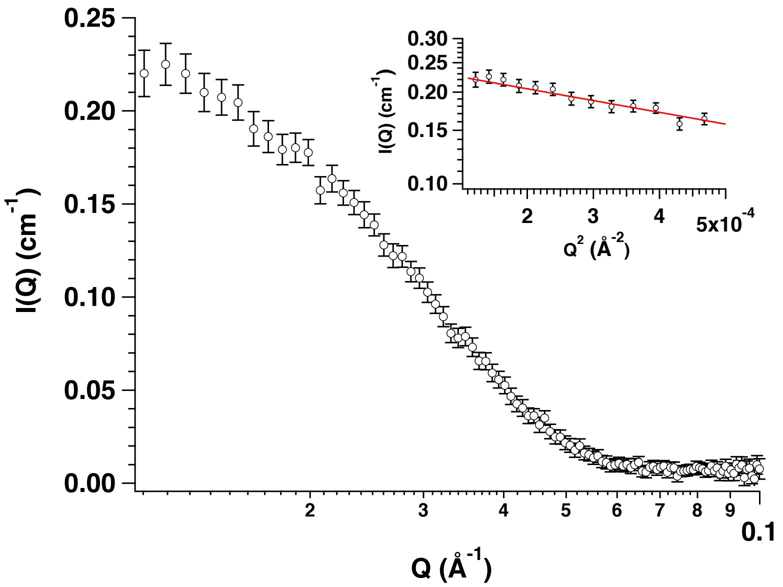

Fig. S14. SANS profile from OLG(0) at the concentration of 0.45 mg/mL in full D_2O (99.6% D_2O ratio) buffer and the inset indicates its Guinier plot.

This figure is prepared by the usage of IGOR Pro 6.34A (https://www.wavemetrics.com/forum/news-and-announcements/igor-634a-now-shipping).

The procedure of time-resolved SANS measurement for both dilute and dense samples

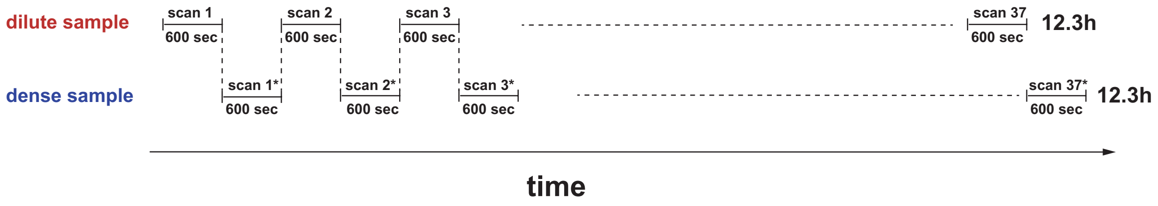

Fig. S15. The schematic picture of procedure of time-resolved SANS measurement for both dilute and dense samples.

This figure is prepared by the usage of Adobe Illustrator CC 2015.2.1 (19.2.1) (https://www.adobe.com/jp/products/illustrator.html).
